# Impact of the “atherosclerotic pabulum” on in‐hospital mortality for SARS‐CoV‐2 infection. Is calcium score able to identify at‐risk patients?

**DOI:** 10.1002/clc.23809

**Published:** 2022-03-30

**Authors:** Valeria Pergola, Giulio Cabrelle, Marco Previtero, Andrea Fiorencis, Giulia Lorenzoni, Carlo Maria Dellino, Carolina Montonati, Saverio Continisio, Elisa Masetto, Donato Mele, Martina Perazzolo Marra, Chiara Giraudo, Giulio Barbiero, Giorgio De Conti, Giovanni Di Salvo, Dario Gregori, Sabino Iliceto, Raffaella Motta

**Affiliations:** ^1^ Department of Cardiac, Vascular, Thoracic Sciences and Public Health University of Padua Padua Italy; ^2^ Department of Medicine, Institute of Radiology University of Padua Padua Italy; ^3^ Unit of Biostatistics, Epidemiology and Public Health, Department of Cardiac, Thoracic, Vascular Sciences, and Public Health University of Padua Padua Italy; ^4^ Radiology Unit Padova University Hospital Padua Italy; ^5^ Department of Women's and Children's Health University of Padua Padua Italy; ^6^ Unit of Advanced and Translational Diagnostics, Department of Medicine University of Padua Padua Italy

**Keywords:** cardiovascular risk, chest computed tomography, coronary calcium score, SARS‐CoV‐2 infection

## Abstract

**Background:**

Although the primary cause of death in COVID‐19 infection is respiratory failure, there is evidence that cardiac manifestations may contribute to overall mortality and can even be the primary cause of death. More importantly, it is recognized that COVID‐19 is associated with a high incidence of thrombotic complications.

**Hypothesis:**

Evaluate if the coronary artery calcium (CAC) score was useful to predict in‐hospital (in‐H) mortality in patients with COVID‐19. Secondary end‐points were needed for mechanical ventilation and intensive care unit admission.

**Methods:**

Two‐hundred eighty‐four patients (63, 25 years, 67% male) with proven severe acute respiratory syndrome coronavirus 2 (SARS‐CoV‐2) infection who had a noncontrast chest computed tomography were analyzed for CAC score. Clinical and radiological data were retrieved.

**Results:**

Patients with CAC had a higher inflammatory burden at admission (d‐dimer, *p* = .002; C‐reactive protein, *p* = .002; procalcitonin, *p* = .016) and a higher high‐sensitive cardiac troponin I (HScTnI, *p* = <.001) at admission and at peak. While there was no association with presence of lung consolidation and ground‐glass opacities, patients with CAC had higher incidence of bilateral infiltration (*p* = .043) and higher in‐H mortality (*p* = .048). On the other side, peak HScTnI >200 ng/dl was a better determinant of all outcomes in both univariate (*p* = <.001) and multivariate analysis (*p* = <.001).

**Conclusion:**

The main finding of our research is that CAC was positively related to in‐H mortality, but it did not completely identify all the population at risk of events in the setting of COVID‐19 patients. This raises the possibility that other factors, including the presence of soft, unstable plaques, may have a role in adverse outcomes in SARS‐CoV‐2 infection.

## 1 INTRODUCTION

Since December 2019, severe acute respiratory syndrome coronavirus 2 (SARS‐CoV‐2) infected more than 118 million people worldwide^1^ and it was declared a pandemic by World Health Organization on March 11, 2020.

Although the primary cause of death in COVID‐19 infection is respiratory failure, there are evidence that cardiac manifestations may contribute to overall mortality and can even be the primary cause of death.^2^ More importantly, it is recognized that COVID‐19 is associated with a high incidence of thrombotic complications^1^ and that the thrombotic diathesis is due to endothelial cell dysfunction.^3^ Of note, while there is a strong evidence that known risk factors for coronary artery disease (CAD), such as age, hypertension, and diabetes, are associated with a poorer prognosis,^2^, ^3^, ^4^, ^5^, ^6^ it has been shown that patients with reduced ventricular function do not have increased mortality compared to controls.^7^ In this context, the coronary artery calcium score (CAC score), an established and validated prognostic indicator of CAD, has been of utmost importance in recognizing patients at high risk of poor outcome.^8^, ^9^ Indeed, there are increasing evidence that plaque characteristics are important in defining accurate cardiovascular risk beyond calcifications.^10^ Therefore, our hypothesis was to verify if CAC per se is able to identify patients at risk of adverse outcomes and in‐hospital (in‐H) death in patients with SARS‐CoV‐2.

## 2 METHODS

### 2.1 Study population

We conducted a retrospective, post hoc analysis of all patients admitted to Padua University Hospital with a confirmed COVID‐19 diagnosis by polymerase chain reaction (PCR) from January 2020 to January 2021. Sample for real‐time PCR was obtained by nasal–oral pharyngeal swab. Exclusion criteria were a history of previous percutaneous coronary artery stenting or coronary bypass surgery, as it may interfere with CAC score calculation. We included patients with known previous CAD who were under medical treatment.

Our population consisted of 284 patients who underwent chest computed tomography (CT) scans because of moderate or severe COVID‐19 infection, according to World Health Organization guidelines.^11^ Baseline demographic, clinical, and laboratory variables (including inflammatory biomarkers) were retrieved from our electronic medical record system. High‐sensitivity cardiac troponin I (HScTnI, cutoff value <16 ng/L) was considered suggestive of acute myocardial damage when its value was at least one above the 99th percentile of the upper reference limit.^12^ A HSc‐TnI higher than 200 ng/dl was calculated as the difference between the abnormal value and the normal value. C‐reactive protein (CRP) was considered normal if the value was <10 mg/L. We considered a cardiovascular complication the first ischemic or thrombotic event during the hospitalization with COVID‐19. Written informed consent was obtained by all participants. The study was conducted according to the guidelines of the Declaration of Helsinki and approved by the Ethics Committee of Padua University (CE 154n). Supporting data are available upon request.

### 2.2 CT scan protocol

All CT scans were performed with a 64‐slice CT system (Aquilion 64; Toshiba) and slice CT system (SOMATOM Sensation; Siemens). A Spiral non‐electrocardiogram (ECG) gated technique during a deep inspiratory breath‐hold was employed (tube voltage 120 kV, tube current power 50–200 mAs,). Images were reconstructed with the following parameters: slice thickness 3 mm, the field of view 250–300 mm, convolution kernel filtering b30f. CAC score was performed on the workstation (Vitrea FX, version 1.0; Vital Images), using CAC score analysis software (VScore; Vital Images). Coronary calcium was defined as an area of at least three contiguous voxels in the axial plane in the course of the coronary artery, with an attenuation cutoff of ≥100 HU.

### 2.3 Calcium score analysis

CAC score was performed offline (Vitrea FX, version 1.0; Vital Images), using CAC score analysis software (VScore; Vital Images). Coronary calcium was defined as an area of at least three contiguous voxels in the axial plane in the course of the coronary artery, with an attenuation cut‐off of ≥100 HU (corresponding to a minimum lesion area >1 mm^2^) in the 3.0 mm reconstruction.^8^

Although the traditional Agatston method for measuring CAC requires ECG‐gated acquisition, a good correlation has been demonstrated between CAC identified on non‐gated CT scans and ordinal scores obtained from gated CT scans.^13^ Patient with Calcium were further stratified according to validated CAC score thresholds (1–100: mild; 101–400: moderate; >400: severe)^8^ and to the cutoff point of 10 (Table 1).^14^

**Table 1:** Calcium score according to different classifications

Variable	*N*
Total	284
CAC score 0	142
≥1	142
1–100	46
101–400	39
≥400	57
≤10	151
11–99	37
≥100	96

Abbreviations: CAC, coronary artery calcium; *N*, number of patients.

We evaluated the occurrence of complications including acute coronary syndrome (ACS), embolic events (cerebral or peripheral), pulmonary embolism, myocarditis, pericarditis, acute heart failure, septic shock, severe acute respiratory distress syndrome, acute kidney injury, and deep vein thrombosis. The primary endpoint was in‐H mortality. The secondary endpoint was need for admission to the intensive care unit (ICU) and mechanical ventilation.

### 2.4 Statistical analysis

Descriptive statistics were reported as I quartile/median/III quartile for continuous data and percentages (absolute numbers) for categorical data.

Univariable and multivariable generalized linear models were estimated to assess the effect of baseline variables on the outcomes of interest using the Aranda link function, which was chosen because it was the parametrization that minimized the Bayesian information criterion.^15^ Multivariable model variable selection was made according to the Akaike information criterion.^16^

The marginal effect was computed considering the partial derivatives of the marginal expectation. Results were reported as average marginal effect (AME), 95% confidence interval, and *p*‐value. The AME expresses the change in probability of the event, that is, ICU admission, in‐H mortality, mechanical ventilation.

Analyses were performed with R system^17^ within rms package.^18^

## 3 RESULTS

Two‐hundred‐eighty‐four patients were analysed.

Overall, the median age was 63, 25 years, 67% were males. Demographic, clinical, and laboratory features stratified by CAC status are presented in Table 2. Ordinal CAC score was calculated in 284 patients, 46 patients having mild (1–100), 39 moderate (101–400), and 57 severe (>400) CAC scores. However, we used only dichotomic values for statistical analysis (CAC = 0 was present in 142 patients, CAC ≥ 1 was present in 142 patients) as we did not note any increase in the outcomes or in cardiovascular complications with increased CAC values.

**Table 2:** Clinical characteristics of patients with and without CAC

Variable	CAC = 0 (*N* = 142)	CAC ≥ 1 (*N* = 142)	*p*
Male sex	58%	77%	.001
Age (years)	45.4/54.6/63.3	64.2/72.2/80.8	<.001
Risk factors			
Hypertension	32%	69%	<.001
Diabetes	19%	27%	.094
Smoking	9%	24%	.001
Obesity	20%	20%	.88
Previous CAD	3%	16%	<.001
Chronic kidney disease	7%	11%	.294
Peripheral vasculopathy	6%	12%	.059
Pulmonary hypertension	1%	0%	.156
Chronic broncopneumopathy	5%	5%	1
Previous malignancy	7%	12%	.209
Active malignancy	9%	10%	.666
Laboratory findings			
WBC × mm^3^	3.6/4.8/6.7	3.8/5.5/7.6	.057
Creatinine (mg/dl)	0.7/0.8/1.1	0.7/0.9/1.2	.218
d‐dimer	150/221/467	182/311/661	.002
CRP‐admission (mg/L)	13/44/98	37/69/120	.002
Procalcitonin	0.04/0.06/0.20	0.05/0.12/0.28	.016
SpO_2_	93/96/98	92/95/97	.01
HScTnI admission (ng/L)	2/5/10	7/14/38	<.001
HScTnI peak (ng/L)	2/5/14	7/20/82	<.001
Chest involvement			
Lung consolidation	64%	66%	.673
GGO	78%	87%	.055
Bilateral involvement	81%	90%	.043
Complications			
All cardiovascular complications	24%	41%	.004
ACS	9%	22%	<.001
Major embolic event	1%	4	.194
Pulmonary embolism	4%	9%	.088
Myocarditis	1%	1%	NA
Pericarditis	6%	10	.348
Acute heart failure	4%	9%	.041
Septic shock	3%	5%	.353
Severe ARDS	10%	12%	.572
Acute kidney injury	5%	10%	.153
DVT	10%	18%	.055
Treatment			
Antibiotic use	95%	95%	.967
Antiviral use	30%	40%	.101
Hydroxychloroquine	34%	28%	.282
Corticosteroids	54%	63%	.105
Tocilizumab	5%	6%	.638
Plasma	14%	17%	.553
Outcomes			
In‐H mortality	7%	14%	.048
ICU	20%	24%	.442
Days in ICU	6/14/23	7/16/32	.354
Mechanical ventilation	17%	20%	.509

*Note*: Data are percentages for categorical variables and I quartile/median/III quartile for continuous variables.

Abbreviations: ACS, acute coronary syndrome; ARDS, acute respiratory distress syndrome; CAC, coronary artery calcium, CAD, coronary artery disease; CRP, C‐reactive protein; DVT, deep vein thrombosis; GGO, ground‐glass opacification; HScTnI, high‐sensitivity cardiac troponin I; ICU, intensive care unit; in‐H, in‐hospital; NA, not applicable; WBC, white blood count.

As expected, factors associated with CAC were male sex, age, hypertension, diabetes, smoke, and previous CAD. Of note patients with CAC had a higher inflammatory burden at admission (d‐dimer, CRP, and procalcitonin) and higher HScTnI at admission and at peak. While there was no association with the presence of lung consolidations, patients with CAC had a higher incidence of bilateral pulmonary involvement and a trend towards worse GGO.

In‐H mortality was associated with CAC. Nevertheless, it did not increase for each point increment in CAC. As expected, in‐H mortality was associated with age but also with hypertension, hyperlipidaemia, obesity, and previous CAD. It was indeed related to lung consolidations and with a higher inflammatory response (Table 3A, 3B, 3C, 4). Of note, peak HScTnI >200 ng/dl was positively associated with in‐H mortality both at univariable and multivariable analysis.

**Table 3A:** Outcome analysis: In‐H mortality

Variable	*0* (*N* = 249)	*1* (*N* = 29)	Average marginal effect (AME)	*p*	Lower	Upper
CAC	48%	68%	0.0725	.027	0.0079	0.1371
Age (years)	51.4/61.9/74.1	67.7/74.8/83.7	0.0056	<.001	0.0031	0.0082
Male sex	66%	79%	−0.0571	.078	−0.1205	0.0063
Hypertension	46%	83%	0.1364	<.001	0.0653	0.2076
Diabetes	23%	21%	−0.0142	.717	−0.0912	0.0627
Smoking	15%	24%	0.0643	.24	−0.043	0.1715
Obesity	19%	23%	0.0198	.6792	−0.074	0.1136
Dyslipidemia	27%	52%	0.1111	.013	0.0233	0.1989
WBC	3.785/5.130/7.030	3.330/4.270/7.860	−0.0003	.942	−0.009	0.0083
Creatinine (mg/dl)	0.7/0.840/1.100	0.7/1.0/1.2	−0.0038	.548	−0.0162	0.0086
CRP admission (mg/L)	20/59/96	60/98/130	0.0006	.008	0.0002	0.0011
Procalcitonin	0.40/0.08/0.20	0.09/0.20/0.40	0.0178	.308	−0.0164	0.052
Saturation O_2_%	93/96/97	88/91/94	−0.0093	.003	−0.0154	−0.0032
HScTnI admission	3.00/7.00/18.00	14.00/29.00/107.75	0	.981	−0.0003	0.0003
Lung consolidation	63%	82%	0.0805	.015	0.0155	0.1455
GGO	81%	89%	0.0527	.091	−0.0083	0.1138
Bilateral involvement	86%	93%	0.0575	.243	−0.0391	0.154
Antibiotic use	94%	100%	0.1038	<.001	0.0691	0.1386
Antiviral use	38%	22%	−0.0607	.096	−0.1322	0.0109
Hydroxychloroquine	31%	37%	0.0235	.549	−0.0535	0.1006
Corticosteroids	56%	78%	0.0799	.011	0.0183	0.1415
Tocilizumab	6%	4%	−0.0383	.618	−0.1886	0.112
Plasma	16%	11%	−0.0341	.374	−0.1094	0.0411
Days in ICU	6/11/20	12/20/35	0.0082	.0123	0.0018	0.0146
d‐dimer >1000	8%	34%	0.2663	.004	0.0847	0.4480
HScTnI‐peak 34–200	12%	31%	0.2245	.006	0.0658	0.3833
HScTnI‐peak >200	5%	62%	0.6046	<.001	0.4039	0.8053
Previous CAD	9%	24%	0.1606	.0491	0.0007	0.3205
Chronic kidney disease	9%	8%	0.05415	.3761	−0.06575	0.174
Peripheral vasculopathy	9%	8%	−0.01054	.8781	−0.1452	0.1241
Pulmonary hypertension	1%	0%				
Chronic broncopneumopathy	4%	12%	0.1448	.1766	−0.06523	0.3549
Previous malignancy	9%	16%	0.07429	.3949	−0.09685	0.2454
Active malignancy	9%	16%	0.06778	.3147	−0.06436	0.1999
Multivariate analysis: AME, *p* (*p*‐value), and lower and upper bound of the 95% confidence interval						

Variable	AME	*p*	Lower	Upper
Antibiotic treatment	0.1052	<.001	0.0537	0.1568
Peak HScTnI 34–200	0.2398	.010	0.0582	0.4214
Peak HScTnI >200	0.5792	<.001	0.4354	0.7231

*Note*: Data are percentages for categorical variables and I quartile/median/III quartile for continuous variables. The table also reports the results of the univariate models, as AME, *p* (*p*‐value), and lower and upper bound of the 95% confidence interval

Abbreviations: CAC, coronary artery calcium; CAD, coronary artery disease; CRP, C‐reactive protein; GGO, ground‐glass opacification; HScTnI, high‐sensitivity cardiac troponin I; ICU, intensive care unit; in H, in hospital; WBC, white blood count.

**Table 3B:** Outcome analysis: ICU admission

Variable	0 (*N* = 219)	1 (*N* = 63)	Average marginal effect (AME)	*p*	Lower	Upper
CAC	49%	55%	0.0385	.495	−0.0721	0.1491
Age (years)	51.450/62.100/76.700	56.500/67.300/73.850	0.0022	.057	−0.0001	0.0046
Male sex	65%	76%	−0.0865	.053	−0.1741	0.0011
Hypertension	46%	67%	0.1458	.001	0.0634	0.2282
Diabetes	21%	25%	0.0394	.52	−0.0804	0.1591
Smoking	14%	25%	0.1478	.056	−0.0038	0.2994
Obesity	20%	20%	0.0029	.9647	−0.1272	0.1330
Dyslipidemia	28%	38%	0.0844	.052	−0.0006	0.1693
WBC × mm^3^	3.7/4.9/6.8	3.9/5.5/10.9	0.0107	.079	−0.0012	0.0226
Creatinine (mg/dl)	0.7200/0.8000/1.0700	0.7300/0.9100/1.2825	−0.0033	.705	−0.0205	0.0138
CRP admission (mg/L)	17/55/89	58/100/160	0.0018	<.001	0.0012	0.0025
Procalcitonin	0.0400/0.0600/0.1525	0.0975/0.2700/0.4825	0.0003	.994	−0.073	0.0736
Saturation O_2_	93/96/97	88/92/95	−0.0213	.001	−0.0333	−0.0092
HScTnI admission (ng/L)	3/6/18	8/14/40	0	.945	−0.0007	0.0008
consolidation	14%	25%	0.1478	.056	−0.0038	0.2994
GGO	79%	94%	0.1836	<.001	0.1008	0.2665
Bilateral infiltration	83%	97%	0.2077	<.001	0.1248	0.2906
Antibiotic use	94%	100%	0.2293	<.001	0.1749	0.2837
Antiviral use	35%	38%	0.0189	.746	−0.0954	0.1332
Hydroxychloroquine	35%	18%	−0.134	.001	−0.2147	−0.0533
Corticosteroids	53%	79%	0.1806	<.001	0.0854	0.2759
Tocilizumab	5%	7%	0.0341	.774	−0.1988	0.267
Plasma transfusion	13%	25%	0.146	.031	0.013	0.279
d‐dimer >1000	10%	18%	0.1670	.028	0.0180	0.3160
Peak HScTnI 34–200	12%	21%	0.2030	.0338	0.0155	0.3905
Peak HScTnI >200	5%	26%	0.4470	<.001	0.2468	0.6471
Previous CAD	9%	13%	0.0666	.4275	−0.0976	0.2311
Chronic kidney disease	8%	14%	0.116	.2873	−0.09765	0.3296
Peripheral vasculopathy	7%	17%	0.1878	.09081	−0.02986	0.4055
Pulmonary hypertension	0%	2%				
Chronic broncopneumopathy	4%	8%	0.1502	.2977	−0.1325	0.433
Previous malignancy	9%	12%	0.05042	.6034	−0.1398	0.2407
Active malignancy	10%	8%	−0.02369	.7462	−0.1672	0.1198
Multivariate analysis: Data are AME, *p* (*p*‐value), and lower and upper bound of the 95% confidence interval						

Variable	AME	*p*	Lower	Upper
Antibiotics	0.2554	<.001	0.1979	0.3129
Bilateral infiltrates	0.1632	.008	0.0426	0.2839
Peak HScTnI 34–200	0.1788	.031	0.0164	0.3412
Peak HScTnI >200	0.3350	.002	0.1273	0.5428
Saturation O_2_	−0.0147	.030	−0.0279	−0.0014

*Note*: Data are percentages for categorical variables and I quartile/median/III quartile for continuous variables. The table also reports the results of the univariate models, as AME, *p* (*p*‐value), and lower and upper bound of the 95% confidence interval.

Abbreviations: CAC, coronary artery calcium; CAD, coronary artery disease; CRP, C‐reactive protein; GGO, ground‐glass opacification; HScTnI, high‐sensitivity cardiac troponin I; ICU, intensive care unit; WBC, white blood count.

**Table 3C:** Outcome analysis: Mechanical ventilation

Variable	0 (*N* = 229)	1 (*N* = 52)	Average marginal effect (AME)	*p*	Lower	Upper
CAC	50%	55%	0.0309	.583	−0.0794	0.1412
Age (years)	51.2/62.2/76.6	57.3/67.0/73.2	0.0022	.026	0.0003	0.0042
Male sex	65%	81%	−0.1088	.007	−0.1877	−0.0299
Hypertension	46%	69%	0.141	<.001	0.0706	0.2115
Diabetes	23%	21%	−0.0135	.798	−0.1168	0.0899
Smoking	14%	25%	0.1159	.13	−0.0341	0.266
Obesity	21%	16%	−0.0429	.4497	−0.1541	0.0683
Dyslipidemia	28%	38%	0.072	.121	−0.0191	0.1631
WBC × mm^3^	3.7/5.1/7.0	3.8/5.0/11.0	0.0066	.199	−0.0035	0.0167
Creatinine (mg/dl)	0.7225/0.8200/1.0675	0.7000/0.9700/1.3250	−0.0019	.821	−0.0182	0.0145
CRP‐admission (mg/L)	18/56/91	59/100/160	0.0015	<.001	0.0009	0.0021
Procalcitonin	0.04/0.65/0.16	0.10/0.27/0.49	0.001	.975	−0.0638	0.0658
Saturation O_2_	93/96/97	88/92/95	−0.0174	.001	−0.0279	−0.0068
HScTnI admission	3/6/20	8.275/14.000/30.000	0	.974	−0.0005	0.0005
Lung consolidations	61%	85%	0.164	.001	0.0635	0.2644
GGO	79%	94%	0.1602	<.001	0.0775	0.2429
Bilateral involvement	83%	98%	0.1928	<.001	0.1154	0.2702
Antibiotic use	94%	100%	0.1917	<.001	0.1475	0.236
Antiviral use	36%	33%	−0.0189	.721	−0.1225	0.0848
Hydroxychloroquine	35%	14%	−0.1487	<.001	−0.215	−0.0824
corticosteroids	54%	80%	0.1638	<.001	0.0793	0.2483
Tocilizumab	6%	4%	−0.0606	.507	−0.2398	0.1186
Plasma transfusion	13%	27%	0.1614	.006	0.0459	0.2769
d‐dimer 500–1000	16%	18%	0.0421	.064	−0.0839	0.1681
d‐dimer >1000	9%	20%	0.1709	.020	0.0271	0.3147
Peak HScTnI 34–200	12%	20%	0.1481	.0863	−0.0212	0.3175
Peak HScTnI >200	6%	28%	0.4009	<.001	0.1859	0.6159
Previous CAD	10%	10%	−0.0021	.9796	−0.1605	0.1564
Chronic kidney disease	8%	16%	0.156	.1019	−0.03094	0.3429
Peripheral vasculopathy	8%	14%	0.1006	.2402	−0.06724	0.2684
Pulmonary hypertension	0%	2%				
Chronic broncopneumopathy	5%	6%	0.03804	.751	−0.1969	0.273
Previous malignancy	9%	12%	0.04953	.582	−0.1268	0.2259
Active malignancy	9%	10%	0.01631	.8609	−0.1661	0.1987
Multivariate analysis: Data are AME, *p* (*p*‐value), and lower and upper bound of the 95% confidence interval						

Variable	AME	*p*	Lower	Upper
No antibiotic use	0.2117	<.001	0.1570	0.2664
Bilateral infiltration	0.1634	.004	0.0537	0.2731
Lung consolidations	0.1348	.003	0.0472	0.2225
CRP	0.0011	<.001	0.0005	0.0018
Hydroxychloroquine	−0.1624	<.001	−0.2505	−0.0743
Peak HScTnI 34–200	0.1788	.031	0.0164	0.3412
Peak HScTnI >200	0.3350	.002	0.1273	0.5428

*Note*: Data are percentages for categorical variables and I quartile/median/III quartile for continuous variables. The table also reports the results of the univariate models, as AME, *p* (*p*‐value), and lower and upper bound of the 95% confidence interval.

Abbreviations: CAC, coronary artery calcium; CAD, coronary artery disease; CRP, C‐reactive protein; GGO, ground‐glass opacification; HScTnI, high‐sensitivity cardiac troponin I; ICU, intensive care unit; WBC, white blood count.

CAC was not associated with the need of ICU admission and mechanical ventilation (Table 3A, 3B, 3C, 4), whereas it appears that HScTnI >200 ng/L was associated with both.

Older age, hypertension, hyperlipidemia, and smoking were positively associated with in‐H mortality, need for ICU, and mechanical ventilation, also when considered as composite outcomes. The same increasing trend across the groups was observed for laboratory data at admission (CRP and HScTnI peak). In particular, CRP and HScTnI >200 ng/L remained positively associated with the composite outcome also in the multivariable model (Table 3B, 4).

**Table 4:** Composite outcome: Death, ICU admission, and mechanical ventilation

Variable	0 (*N* = 206)	1 (*N* = 74)	Average marginal effect (AME)	*p*	Lower	Upper
CAC	48%	57%	0.067	.247	−0.0464	0.1805
Age	51.250/61.850/75.025	58.075/68.650/76.675	0.0049	<.001	0.0022	0.0075
Male sex	65%	76%	−0.0947	.077	−0.1999	0.0104
Hypertension	44%	68%	0.1819	<.001	0.0801	0.2838
Diabetes	22%	24%	0.0276	.666	−0.0979	0.1532
Smoking	14%	24%	0.151	.046	0.003	0.299
Obesity	20%	18%	−0.0205	.7703	−0.1583	0.1172
Dyslipidemia	26%	42%	0.1497	.016	0.0278	0.2715
WBC	3.7400/4.9000/6.7300	3.7950/5.4800/10.7375	0.0121	.067	−0.0009	0.0252
Creatinine (mg/dl)	0.720/0.800/1.065	0.760/0.920/1.300	−0.0044	.475	−0.0165	0.0077
CRP admission (mg/L)	16.00/50.50/87.25	59.25/100.00/157.50	0.0021	<.001	0.0013	0.0029
Procalcitonin	0.0400/0.0600/0.1500	0.0800/0.2300/0.4600	0.0397	.43	−0.0589	0.1383
Saturation O_2_	94/96/97	88/92/95	−0.0276	.001	−0.0439	−0.0113
Consolidation	61%	79%	0.1665	.001	0.0713	0.2617
GGO	79%	90%	0.1555	.014	0.0311	0.28
Bilateral infiltration	83%	96%	0.2184	<.001	0.1167	0.3201
Antibiotics	93%	100%	0.2727	<.001	0.2308	0.3147
Antiviral	36%	35%	−0.014	.815	−0.1316	0.1035
Hydroxychloroquine	35%	22%	−0.1108	.029	−0.2103	−0.0113
Corticosteroids	52%	78%	0.2044	<.001	0.0968	0.312
Tocilizumab	6%	6%	−0.0095	.941	−0.2635	0.2444
Plasma	14%	22%	0.1243	.119	−0.0321	0.2808
d‐dimer 500–1000	14%	22%	0.1585	.054	−0.0029	0.3199
d‐dimer >1000	7%	23%	0.3433	<.001	0.1654	0.5211
Peak HScTnI 34‐200	10%	24%	0.3155	.002	0.1205	0.5105
Peak HScTnI>200	3%	30%	0.6375	<.001	0.4735	0.8014
Previous CAD	8%	15%	0.1398	.1938	−0.0711	0.3508
Chronic kidney disease	8%	13%	0.114	.2753	−0.09083	0.3189
Peripheral vasculopathy	7%	16%	0.1842	.07392	−0.01782	0.3862
Pulmonary Hypertension	0%	1%				
Chronic broncopneumopathy	3%	10%	0.2568	.03475	0.0184	0.4951
Previous malignancy	9%	11%	0.05769	.4664	−0.09756	0.2129
Active malignancy	9%	11%	0.05769	.5073	−0.1128	0.2282
Multivariate analysis: Data are AME, *p* (*p*‐value), and lower and upper bound of the 95% confidence Interval						

Variable	AME	*p*	Lower	Upper
Antibiotic	0.2865	<.001	0.2258	0.3472
CRP	0.0013	<.001	0.0007	0.0018
Peak HScTnI 34–200	0.2439	.005	0.0737	0.4140
Peak HScTnI>200	0.4801	<.001	0.2891	0.6711
Saturation O_2_	−0.0154	.022	−0.0286	−0.0023
Chronic broncopneumopathy	0.2568	.03475	0.0184	0.4951

*Note*: Data are percentages for categorical variables and I quartile/median/III quartile for continuous variables. The table also reports the results of the univariable models, as AME, *p* (*p*‐value), and lower and upper bound of the 95% confidence Interval.

Abbreviations: CAC, coronary artery calcium; CAD, coronary artery disease; CRP, C‐reactive protein; GGO, ground‐glass opacification; HScTnI, high‐sensitivity cardiac troponin I; ICU, intensive care unit; WBC, white blood count.

## 4 DISCUSSION

Data from multiple cohorts shows that CAC effectively stratifies patients for long‐term all‐cause and cardiovascular mortality better than traditional risk factors.^11^, ^19^, ^20^, ^21^, ^22^ On the contrary, the effects of CAC on in‐H mortality due to other causes, like sepsis, have been less explored.

The main finding of our study is the presence of calcium, was related to peak HScTnI. Peak HScTnI was linked with all the endpoints. CAC was associated with a higher rate of cardiovascular complications which was likely related to the increase in mortality. This association was not observed after correcting for traditional risk factors linked to worse COVID‐19 outcomes such as age, diabetes, hypertension, and hyperlipidaemia.

### 4.1 Comparison with previous studies

Our data are partially in agreement with Slipchuck et al.,^23^ who compared baseline characteristics and outcomes of patients admitted with COVID‐19 who had a CT study with patients who did not have a CT performed. Their patients had no previous history of percutaneous coronary intervention or coronary artery bypass grafting. They showed that for each point increase in CAC, mortality increased by 8% in 4 months follow‐up. We did not find this association as we only tested in hospital mortality, not follow‐up. In their study, CTs were obtained up to 5 years before index hospitalization, while in our study CTs were all done during admission to exclude CAC variation in our patients.

Gupta et al.^24^ demonstrated that CAC stratifies septic patients for cardiovascular complications better than traditional risk factors. CAC score was also evaluated in COVID‐19 patients in smaller trials. Our data confirm the findings from an Italian cohort of patients (332 patients, 68 deaths and mortality of 20.5%) who found a correlation between CAC on admission and mortality that did not persist after multivariable correction.^25^ Compared to our study, patients in the study by Ferrante et al.^25^ had significantly lower comorbidities with less diabetes and hyperlipidaemia and lower incidence of CAC (CAC ≥ 1 of 43.9% vs. 50% in our study) and a lower incidence of events. Other small studies suggested a correlation of CAC and adverse events such as mechanical ventilation/extra‐ or death.^26^, ^27^, ^28^ Our findings did not confirm these studies' hypothesis as we found no correlation between CAC and need for mechanical ventilation or admission in intensive care.

In the study by Scoccia et al.,^29^ they spotted that clinical and subclinical CAD assessed by CAC score on a routine ECG nongated chest CT are associated with in‐H mortality and myocardial infarction/cerebrovascular accident. They also discovered that traditional cardiovascular risk factors are not independently associated with COVID‐19 in‐H mortality when the extent and presence of coronary atherosclerosis is considered. On the contrary, in our study, on the multivariable analysis emerged that high peak troponin was significantly correlated with in hospital mortality and other outcomes, indicating that CAC does not completely identify patients at risk of cardiovascular events because probably it does not reveal soft, unstable plaques that are more sensitive to external stresses.^30^

### 4.2 Limitations of CAC score

Studies have shown that there is an increase in noncalcified plaque volumes in ACS patients. Moreover, when coronary computed tomography angiography plaque features are accounted for, patients with widespread nonobstructive CAD had similar event rates compared with patients with localized obstructive disease, suggesting that plaque characteristics are important in defining accurate cardiovascular risk beyond calcifications.^30^

The main finding of our research is that CAC alone does not completely identify all the population at risk of cardiovascular events in the setting of COVID‐19 patients. On the other hand, HscTnI was a better determinant of outcomes.^10^, ^29^ Therefore, it could be hypothesized that other factors, including the presence of soft plaques, may be a substratum where hypoxemia, systemic inflammation, endothelial injury triggered by direct virus activity through angiotensin‐converting enzyme 2 endothelial receptor, followed by platelet activation triggers cardiovascular events,^31^ thus increasing the rate of adverse outcomes.

## 5 CONCLUSION

Our findings demonstrated that peak HScTnI is linked with all the endpoints in COVID‐19 patients. CAC score was not, per se, the strongest marker for the considered endpoints. This arises the possibility CAC score may slightly underestimate the risk of adverse events. These findings support the conduct of larger trials on cardiovascular disease potentially in other infectious and inflammatory diseases.

### 5.1 Limitations

The study's inclusion criteria of infected patients who had a chest CT selected a higher‐risk population, reflected in the higher mortality rate. We did not consider in our analysis the impact of CAC in patients with milder infection.

## CONFLICTS OF INTEREST

The authors declare no conflicts of interest.

## AUTHOR CONTRIBUTIONS

**Valeria Pergola**: Conceptualization, methodology, and writing – original draft preparation. **Giulio Cabrelle**: Conceptualization, methodology, and writing – original draft preparation. **Giulio Barbiero and Andrea Fiorwncis**: Investigation and methodology. **Chiara Giraudo and Marco Previtero**: Data curation and software. **Carlo M. Dellino, Carolina Montonati, and Saverio Continisio**: Visualization and investigation. **Donato Mele and Martina Perazzolo Marra**: Supervision: **Giulia Lorenzoni and Elisa Masetto**: Software and formal analysis. **Giovanni Di Salvo and Dario Gregorio**: Formal analysis and validation: **Raffaella Motta and Sabino Iliceto**: Writing – reviewing and editing (equally contributed).

## Data Availability

The data that support the findings of this study are available on request from the corresponding author. The data are not publicly available due to privacy or ethical restrictions.
